# Association of Advanced Airway Management in Preference to Intravenous Adrenaline on Neurological Outcomes Following Out-of-Hospital Cardiac Arrest

**DOI:** 10.7759/cureus.59926

**Published:** 2024-05-08

**Authors:** Yasutaka Koga, Motoki Fujita, Takeshi Yagi, Masaki Todani, Takashi Nakahara, Kotaro Kaneda, Ryosuke Tsuruta

**Affiliations:** 1 Advanced Medical Emergency and Critical Care Center, Yamaguchi University Hospital, Ube, JPN

**Keywords:** pre-hospital emergency medicine, emergency medical service, out-of-hospital cardiac arrest, advanced airway management, adrenalin

## Abstract

Aim: To examine the preference for advanced airway management (AAM) or intravenous adrenaline administration (IVAd) provided by emergency medical services (EMS) for out-of-hospital cardiac arrest (OHCA) with shockable or nonshockable rhythms.

Methods: We conducted a retrospective analysis of a nationwide cohort of OHCA patients in Japan. Adult patients with witnessed collapse who were provided AAM and/or IVAd by EMS between June 2014 and December 2019 were divided into the AAM preferred group and IVAd preferred group, according to the initial advanced EMS intervention. The rates of favorable neurological outcomes (cerebral performance category 1 or 2 after 30 days) were compared between groups of patients with initial shockable or nonshockable rhythms.

Results: We analyzed 1365 and 9733 patients with initial shockable and nonshockable rhythms, respectively. Of these patients, 1033 (75.7%) with shockable and 7844 (80.6%) with nonshockable rhythms, respectively, were assigned to the AAM preferred group. Favorable neurological outcomes were significantly more frequent in the AAM preferred group than in the IVAd preferred group in patients with a shockable rhythm (13.6% vs 9.3%, respectively; P = 0.039), but not in those with a nonshockable rhythm (1.0% vs 0.8%, respectively; P = 0.509). Preferred AAM was independently associated with a higher probability of favorable neurological outcomes in patients with a shockable rhythm (adjusted odds ratio 1.66, 95% confidence interval 1.08-2.53, P = 0.020), but not in patients with a nonshockable rhythm.

Conclusions: AAM provided by EMS in preference to IVAd was associated with the favorable neurological outcomes of OHCA patients with shockable rhythms.

## Introduction

Out-of-hospital cardiac arrest (OHCA) is an important problem, with extremely high mortality and morbidity. In Japan, over 120,000 patients with OHCA are transferred to hospital by ambulance each year, and survival and a favorable neurological outcome at day 30 are achieved in only 11.4-13.9% and 7.2-9.1% of these patients, respectively, even when their collapse is witnessed [1].

To achieve better outcomes in patients with OHCA, early and high-quality basic life support (BLS), such as chest compression and defibrillation with a public automated external defibrillator (AED), is extremely important. Advanced life support (ALS) is provided to patients who do not achieve a return of spontaneous circulation (ROSC) with BLS. ALS includes interventions such as intravenous adrenaline administration (IVAd) and advanced airway management (AAM) with a supraglottic airway device (SGA) or endotracheal intubation. Although these advanced interventions were formerly only performed by medical professionals in hospitals, they are currently performed by trained emergency medical services (EMS) personnel in prehospital settings.

Although the effectiveness of these prehospital advanced interventions by EMS personnel has not been established, their earlier administration may increase their effectiveness [2-5]. However, limited EMS personnel restricts the concurrent administration of these interventions in prehospital settings. Therefore, it is important to consider which AAM or IVAd performed by EMS personnel in prehospital settings is the most preferable intervention. To examine this clinical question, we conducted a retrospective analysis of data on a Japanese nationwide prospective cohort of OHCA patients.

## Materials and methods

Study design

This study was a retrospective analysis of data from the Japanese Association for Acute Medicine Out-of-Hospital Cardiac Arrest (JAAM-OHCA) registry, a nationwide multicenter prospective cohort in Japan [6]. This cohort included patients with OHCA who were transferred directly to 101 hospitals in Japan. The registry was approved by the ethics committee of each participating institution.

Prehospital resuscitation in Japan

In Japan, ambulances are dispatched 24 hours a day in response to emergency calls. Three EMS personnel, usually including some emergency life-saving technicians (ELSTs) who are trained in several medical interventions, travel in the ambulance. Emergency physicians and nurses are also dispatched to prehospital settings by a doctor delivery system in some regions. For patients with OHCA, these EMS personnel provide BLS with chest compression, ventilation with a bag-valve-mask apparatus, and defibrillation with an AED for those with a shockable rhythm. Mechanical chest compression devices may be used instead of manual chest compression by EMS personnel, if available. Advanced EMS interventions (AAM and IVAd) can only be provided by ELSTs. All ELSTs can provide AAM with an SGA for patients whose airways are not maintained manually, with or without cardiac arrest. Especially trained ELSTs can also provide tracheal intubation and IVAd for patients with cardiac arrest according to a physician’s instructions by phone. In Japan, because EMS have no legal authority to terminate resuscitation in OHCA patients with no clear postmortem change (e.g., rigor mortis, livor mortis, or putrefaction), almost all OHCA patients treated by EMS, with or without ROSC, are immediately transferred to hospital.

Data collection

The data from the JAAM-OHCA registry included prehospital resuscitation data from the All-Japan Utstein Registry of the Fire and Disaster Management Agency of Japan and in-hospital data collected prospectively by each institution participating in the JAAM-OHCA registry. From these data, we obtained the following patient information: age, sex, cause of arrest, witness of collapse, layperson bystander cardiopulmonary resuscitation (CPR), documented initial rhythm, time course of resuscitation, prehospital intervention (defibrillation, AAM, or IVAd), and outcome. The primary outcome in this study was favorable neurological outcomes defined as a Cerebral Performance Category (CPC) score of 1 or 2 at day 30. Secondary outcomes included pre- and in-hospital ROSC, time to initial ROSC, and survival at day 30.

Participants

We analyzed adult patients with OHCA who underwent attempted resuscitation and were provided AAM and/or IVAd by ELSTs in a prehospital setting between June 2014 and December 2019. To clarify the time dependence of these interventions, we included only those patients whose collapse was witnessed. Patients were excluded if they were treated in a prehospital setting by physicians dispatched by a doctor delivery system or if they had incomplete data. Additionally, patients who received both advanced EMS interventions at the same time (i.e. with a time interval between interventions of less than one minute) were excluded because the timing of interventions was recorded in minutes, making it impossible to determine their preferred intervention in these cases. Since ELSTs can provide AAM with an SGA not only to patients with sustained cardiac arrest but also to those with ROSC, AAM provided with an SGA in resuscitated patients could not be distinguished from AAM provided during recurrent cardiac arrest in our data. Therefore, we also excluded those patients whose initial advanced EMS intervention was provided after initial ROSC. Patients who received extracorporeal CPR were included.

Statistical analysis

We divided the patients into two groups, the AAM preferred group and the IVAd preferred group, according to the advanced EMS intervention initially provided. Patients who were provided only AAM or IVAd were included in the corresponding group. The background characteristics of the patients (e.g. age, sex), the time course of resuscitation, and the outcomes were compared between the two groups. Variables are shown as medians (interquartile ranges (IQR)) or numbers (percentages). Univariate analyses employed a χ2 test for categorical variables and the Mann-Whitney U test for continuous variables. Multivariable logistic regression analyses were performed to determine the adjusted odds ratio (OR) for preferred AAM compared with preferred IVAd for outcomes, after adjustment for the variables age, sex, cause of cardiac arrest, layperson bystander CPR, response time, and no-flow time. Because the recommendations for IVAd in current guidelines differ for shockable and nonshockable rhythms [7], we performed all analyses separately in patients with an initial shockable rhythm and those with a nonshockable rhythm. P < 0.05 was considered statistically significant. All analyses were performed using Statistical Package for the Social Sciences (IBM SPSS Statistics for Windows, IBM Corp., Version 22.0, Armonk, NY).

## Results

Patients and their characteristics

**Figure 1:**
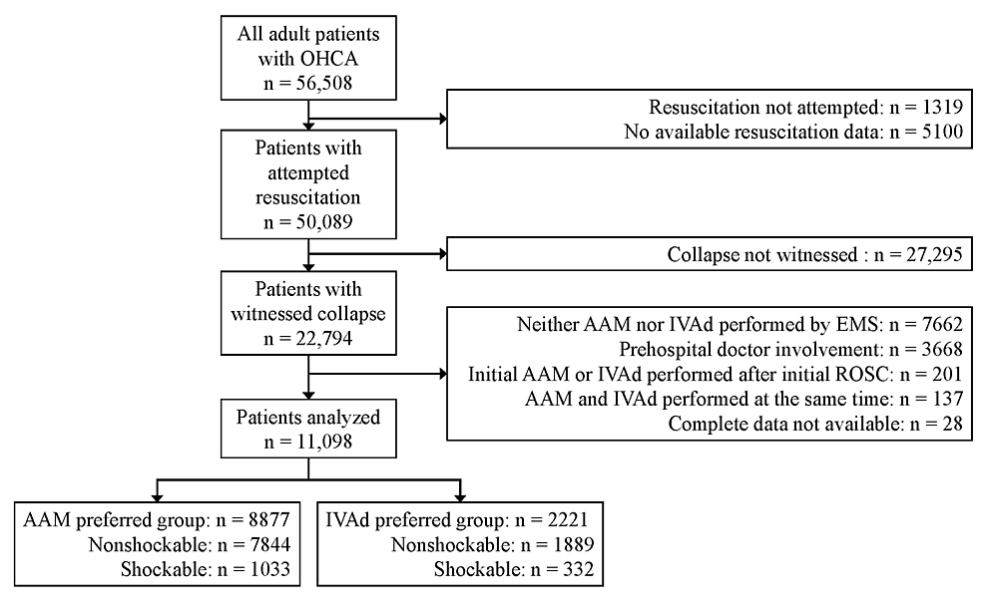
Patient flowchart AAM: advanced airway management; EMS: emergency medical services; IVAd: intravenous adrenaline administration; OHCA: out-of-hospital cardiac arrest; ROSC: return of spontaneous circulation

Of 56,508 adult patients with OHCA, 11,098 patients were analyzed in the present study (Figure 1). The most frequent reason for exclusion was the lack of a witness or the lack of advanced EMS intervention. Among the patients analyzed, 1365 (12.3%) had an initial shockable rhythm. AAM was provided as the initial advanced EMS intervention for 1033 patients (75.7%) with and 7844 patients (80.6%) without an initial shockable rhythm. The background characteristics of the patients are shown in Table 1. On average, the patients were 77 (66-85) years old, and 4285 patients (38.6%) were female. The cause of cardiac arrest was diagnosed as cardiogenic in 6100 patients (55.0%), with a significantly higher prevalence in patients with an initial shockable rhythm than in those without (1241 patients (90.9%) vs 4859 patients (49.9%), respectively; P < 0.001). Layperson bystander CPR was provided to 4976 patients (44.8%), and ventilation by a layperson bystander was provided to only 925 patients (8.3%). Overall, the median (IQR) duration from witnessed collapse to the emergency call and that from the emergency call to EMS arrival at the scene were 1 min (-3-4) and 8 min (7-10), respectively. These background characteristics did not differ significantly between the AAM and IVAd preferred groups, except that there were more females and nonmedical causes of cardiac arrest and a shorter response time in the AAM preferred group than in the IVAd preferred group among patients with an initial nonshockable rhythm. The no-flow time was shorter in the AAM preferred group than in the IVAd preferred group, with a slight but statistically significant difference (4 min (IQR 0-10) vs 5 min (IQR 1-10), respectively; P = 0.004).

**Table 1:** Background characteristics of patients Values are shown as numbers (percentages) of patients or medians (interquartile ranges). † Time from EMS call to EMS arrival at the scene. ‡ Time from witnessed collapse to start of CPR. AAM: advanced airway management; AED: automated external defibrillator; CPR: cardiopulmonary resuscitation; EMS: emergency medical services; IVAd: intravenous adrenaline administration

	Nonshockable				Shockable		
	AAM preferred	IVAd preferred	P value		AAM preferred	IVAd preferred	P value
	(n = 7844)	(n = 1889)			(n = 1033)	(n = 332)	
Age, year	78 (68-86)	78 (68-85)	0.121		67 (55-76)	65 (53-75)	0.084
Sex (female)	3289 (41.9)	728 (38.5)	0.007		213 (20.6)	55 (16.6)	0.106
Initial cardiac rhythm			0.185				0.611
Ventricular fibrillation	‐	‐			1013 (98.1)	327 (98.5)	
Pulseless ventricular tachycardia	‐	‐			20 (1.9)	5 (1.5)	
Asystole	3791 (48.3)	869 (46)			‐	‐	
Pulseless electrical activity	3596 (45.8)	902 (47.8)			‐	‐	
Undetermined	457 (5.8)	118 (6.2)			‐	‐	
Cause of cardiac arrest			0.029				0.299
Cardiogenic	3889 (49.6)	970 (51.3)			934 (90.4)	307 (92.5)	
Other medical	2385 (30.4)	592 (31.3)			78 (7.6)	17 (5.1)	
Nonmedical	1570 (20.0)	327 (17.3)			21 (2.0)	8 (2.4)	
Layperson bystander CPR	3438 (43.8)	824 (43.6)	0.870		552 (53.4)	162 (48.8)	0.141
Chest compression	3233 (41.2)	798 (42.2)	0.415		513 (49.7)	154 (46.4)	0.299
Ventilation	646 (8.2)	144 (7.6)	0.382		110 (10.6)	25 (7.5)	0.098
Public access AED shock delivery	98 (1.2)	32 (1.7)	0.131		56 (5.4)	25 (7.5)	0.157
Time from witnessed collapse to EMS call, min	1 (-3-4)	1 (-4-4)	0.631		1 (0-3)	1 (0-4)	0.819
Response time^†^, min	8 (7-10)	9 (7-11)	<0.001		8 (7-10)	8 (7-10)	0.780
No-flow time^‡^, min	4 (0-10)	5 (0-10)	0.024		5 (1-9)	5 (2-10)	0.054

Prehospital interventions provided by EMS

The prehospital interventions provided by EMS personnel and their time courses in each group are shown in Table 2. The period from EMS arrival to initial advanced EMS intervention was significantly shorter in the AAM preferred group than in the IVAd preferred group (8 min (IQR 5-11) vs 13 min (IQR 9-17), respectively; P < 0.001). However, among patients who were provided both advanced EMS interventions (3828 patients (43.1%) in the AAM preferred group and 585 patients (26.3%) in the IVAd preferred group), the period from the initial intervention to the second intervention was longer in the AAM preferred group than in the IVAd preferred group (6 min (IQR 3-11) vs 3 min (IQR 2-5), respectively; P < 0.001). An SGA was used for prehospital AAM more frequently in the AAM preferred group than in the IVAd preferred group (7148 patients (80.5%) vs 392 patients (67.0%), respectively; P < 0.001). Most patients with an initial shockable rhythm were provided defibrillation by EMS personnel 1 min (IQR 1-2) after EMS arrival, with no significant difference between the two groups. On the contrary, defibrillation by EMS personnel in patients with an initial nonshockable rhythm was significantly less frequent in the AAM preferred group than in the IVAd preferred group (462 patients (5.9%) vs 179 patients (9.5%), respectively; P < 0.001).

**Table 2:** Interventions provided by EMS personnel in a prehospital setting Values are shown as numbers (percentages) of patients or medians (interquartile ranges). AAM: advanced airway management; AED: automated external defibrillator; EMS: emergency medical services; IVAd: intravenous adrenaline administration

	Nonshockable				Shockable		
	AAM preferred	IVAd preferred	P value		AAM preferred	IVAd preferred	P value
	(n = 7844)	(n = 1889)			(n = 1033)	(n = 332)	
Time from EMS arrival to initial EMS advanced intervention, min	8 (5-12)	13 (9-17)	<0.001		7 (5-10)	11 (8-16)	<0.001
AAM performed	7844 (100)	499 (26.4)	<0.001		1033 (100)	86 (25.9)	<0.001
Time from EMS arrival to EMS AAM, min	8 (5-12)	14 (10-19)	<0.001		7 (5-10)	12 (10-17)	<0.001
Type of AAM			<0.001				<0.001
Laryngeal tube	5552 (70.8)	321 (64.3)			782 (75.7)	54 (62.8)	
Laryngeal mask	746 (9.5)	14 (2.8)			68 (6.6)	3 (3.5)	
Endotracheal intubation	1546 (19.7)	164 (32.9)			183 (17.7)	29 (33.7)	
IVAd performed	3398 (43.3)	1889 (100)	<0.001		430 (41.6)	332 (100)	<0.001
Time from EMS arrival to EMS IVAd, min	15 (11-20)	13 (9-17)	<0.001		14 (9-19)	11 (8-16)	<0.001
Both AAM and IVAd performed	3398 (43.3)	499 (26.4)	<0.001		430 (41.6)	86 (25.9)	<0.001
Time between initial and second intervention, min	6 (3-12)	3 (2-5)	<0.001		6 (3-11)	3 (2-6)	<0.001
Shock delivery	462 (5.9)	179 (9.5)	<0.001		1013 (98.1)	329 (99.1)	0.204
Time from EMS arrival to EMS shock, min	12 (6-19)	12 (5-20)	0.625		1 (1-2)	1 (1-2)	0.248
Time from EMS arrival to hospital admission, min	26 (21-32)	27 (21-34)	<0.001		25 (19-31)	25 (20-32)	0.117

Outcomes and their associations with preferred advanced EMS interventions

Table 3 shows the outcomes in both groups. Among patients with an initial nonshockable rhythm, although the overall ROSC rate was equivalent in the two groups, prehospital ROSC was significantly less frequent in the AAM preferred group than in the IVAd preferred group (1026 patients (13.1%) vs 395 patients (20.9%), respectively; P < 0.001) and was achieved later after witnessed collapse than in the IVAd preferred group (33 (IQR 24-42) min vs 29 (IQR 20-41) min, respectively; P < 0.001). On the contrary, overall ROSC among patients with an initial shockable rhythm was significantly more frequent in the AAM preferred group than in the IVAd preferred group (619 patients (59.9%) vs 173 patients (52.1%), respectively; P = 0.012), with no significant time difference. The survival rate at day 30 did not differ significantly between the two groups, regardless of the initial cardiac rhythm. A favorable neurological outcome at day 30 was significantly more frequent in the AAM preferred group than in the IVAd preferred group among patients with an initial shockable rhythm (141 patients (13.6%) vs 31 patients (9.3%), respectively; P = 0.039), but not among the patients with an initial nonshockable rhythm (75 patients (1.0%) vs 15 patients (0.8%), respectively; P = 0.509).

**Table 3:** Outcomes in both groups Values are shown as numbers (percentages) of patients or medians (interquartile ranges). † Favorable neurological outcome was defined as cerebral performance category 1 or 2. AAM: advanced airway management; AED: automated external defibrillator; EMS: emergency medical services; IVAd: intravenous adrenaline administration; ROSC: return of spontaneous circulation

	Nonshockable				Shockable		
	AAM preferred	IVAd preferred	P value		AAM preferred	IVAd preferred	P value
	(n = 7844)	(n = 1889)			(n = 1033)	(n = 332)	
ROSC							
Prehospital	1026 (13.1)	395 (20.9)	<0.001		212 (20.5)	61 (18.4)	0.394
Overall	3546 (45.2)	855 (45.3)	0.965		619 (59.9)	173 (52.1)	0.012
Time from EMS arrival to initial ROSC, min	32 (23-41)	27 (19-39)	<0.001		30 (17-48)	30 (19-52)	0.280
Time from initial EMS advanced intervention to initial ROSC, min	23 (14-31)	12 (6-24)	<0.001		23 (11-38)	17 (7-39)	0.124
Survival at day 30	433 (5.5)	89 (4.7)	0.161		252 (24.4)	68 (20.5)	0.143
Favorable neurological outcome at day 30^†^	75 (1.0)	15 (0.8)	0.509		141 (13.6)	31 (9.3)	0.039

**Figure 2:**
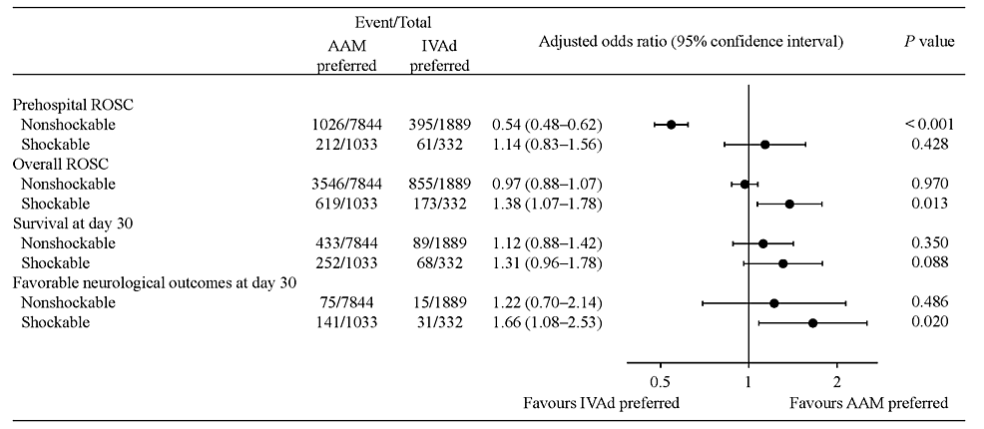
Influence of preferred AAM to IVAd on outcomes Adjustment was made for age, sex, cause of cardiac arrest, layperson bystander cardiopulmonary resuscitation, emergency medical service response time, and no-flow time. AAM: advanced airway management; IVAd: intravenous adrenaline administration; ROSC: return of spontaneous circulation

The adjusted ORs for preferred AAM compared with preferred IVAd for outcomes are shown in Figure 2. Preferred AAM was independently associated with a reduced likelihood of prehospital ROSC among patients with an initial nonshockable rhythm (adjusted OR 0.55, 95%CI 0.48-0.62, P < 0.001), but did not have a significant influence on the overall ROSC of those patients. In contrast, among the patients with an initial shockable rhythm, preferred AAM was independently associated with an increased likelihood of overall ROSC (adjusted OR 1.38, 95%CI 1.07-1.78, P = 0.013), but not of prehospital ROSC. Preferred AAM did not significantly influence survival at day 30, regardless of the initial cardiac rhythm. However, preferred AAM was independently associated with an increased likelihood of a favorable neurological outcome at day 30 among patients with an initial shockable rhythm (adjusted OR 1.66, 95%CI 1.09-2.52, P = 0.029), but not among patients with an initial nonshockable rhythm.

## Discussion

In this study, we have demonstrated that prehospital AAM by ELSTs in preference to IVAd was associated with an increased likelihood of favorable neurological outcomes in patients with an initial shockable rhythm. On the contrary, preferred AAM in patients with an initial nonshockable rhythm was associated with a reduced likelihood of prehospital ROSC, although this intervention did not influence the overall ROSC, survival, or neurological outcome.

Although both AAM and IVAd are included in ALS for cardiac arrest, their benefits in patients with OHCA have not been determined in previous randomized controlled studies [8-11]. However, these advanced interventions are effective if they are provided within the appropriate time frame, because previous observational studies have suggested that the earlier administration of both interventions by EMS personnel is most effective for OHCA patients [2-5]. However, this appropriate timing may differ between patients with shockable and nonshockable rhythms.

In the earliest phase of shockable cardiac arrest, electrical defibrillation is considered sufficient to achieve ROSC without additional intervention in most cases [12]. However, the rate of ROSC by electrical defibrillation decreases over time, because internal oxygen storage is reduced and deleterious metabolites have accumulated. Therefore, advanced interventions, which can improve oxygen delivery and metabolic abnormalities, may be beneficial in patients with shockable cardiac arrest at a specific time interval after collapse. No beneficial effect of IVAd on ROSC during the initial 10 min after the collapse was detected among patients with a shockable rhythm in the post hoc analysis of the PARAMEDIC 2 trial [13]. Moreover, some retrospective studies of in-hospital cardiac arrest with an initial shockable rhythm showed that early IVAd was associated with poor outcomes, independent of delayed defibrillation [14,15]. These results suggest that the premature administration of IVAd for cardiac arrest with a shockable rhythm may not only be ineffective but also harmful, and reduced microvascular blood flow in vital organs due to capillary vasoconstriction was considered to be involved. Although the appropriate timing of AAM in patients with a shockable rhythm is unclear, the harmful effect of IVAd during the very early phase of shockable cardiac arrest may explain our results.

Reduced oxygen storage and accumulated metabolites are common in the early phase of nonshockable OHCA in response to pre-arrest shock or respiratory failure. Therefore, advanced interventions should be provided from the earliest phase. IVAd could increase the likelihood of ROSC among patients with nonshockable OHCA, regardless of the time interval from collapse [10]. In the present study, preferred IVAd led to earlier ROSC in patients with a nonshockable rhythm. However, advanced EMS interventions are insufficient to improve survival or neurological outcomes because of the high probability of irreversible brain damage having occurred by the time of EMS arrival.

This study had several limitations. First, it was a retrospective analysis of a cohort study. Despite adjustment for common prognostic factors, several confounders may have remained. Moreover, the “resuscitation time bias” could affect our results [16]. Certain interventions by EMS personnel, such as IVAd and tracheal intubation, are provided only during cardiac arrest. Consequently, patients who achieve ROSC before these interventions are excluded from the study, potentially leading to a bias wherein patients receiving these interventions may have experienced longer cardiac arrests and worse outcomes. As shown in the present study, the time required to provide the intervention is longer for IVAd than for AAM. Therefore, the IVAd preferred group could been more susceptible to the resuscitation time bias. Although adjustment for the time required to provide an intervention may circumvent this bias, this adjustment could entail serious statistical problems because the time required for the maneuver strongly depended on the advanced interventions themselves. The exclusion or unintentional allocation to another intervention group of patients with failed attempts for the first interventions could also have introduced bias. Secondly, the study included patients with OHCA transferred to a limited number of participating institutions in Japan. The EMS activities for OHCA in prehospital settings vary across countries, and these regional differences, such as in the use of drugs other than adrenaline, patient transfer during resuscitation, or the mean times for response or hospital arrival, may affect the preference for the interventions. Therefore, it is unclear whether our results apply to other countries. Furthermore, because most institutions that participated in this study were tertiary hospitals, there may have been some selection bias. However, because the study focused on prehospital interventions, this may have had only a small effect on our results.

## Conclusions

In this study, AAM provided in preference to IVAd by EMS in a prehospital setting was associated with a significantly higher probability of favorable neurological outcomes at day 30 in OHCA patients with an initial shockable rhythm, compared with IVAd administered in preference to AAM. However, challenges arise in interpreting this result due to differences in the time required for these interventions. Further randomized trials to determine the priorities of advanced EMS interventions are warranted.
